# Comparative Effect of No Finish Line, Heavy Chamfer, and Shoulder Marginal Designs on the Fracture Resistance of Zirconia (Cercon) Ceramic Restoration: An In Vitro Study

**DOI:** 10.7759/cureus.39009

**Published:** 2023-05-14

**Authors:** Sai Govind Gavara, Shashikala Jain, Himanshu Gupta, Suraj Sharma, Pratibha Panwar, Mariyam S Momin

**Affiliations:** 1 Department of Prosthodontics, Crown and Bridge and Oral Implantology, Maharaja Ganga Singh Dental College and Research Centre, Sriganganagar, IND; 2 Department of Periodontics and Oral Implantology, Maharaja Ganga Singh Dental College and Research Centre, Sriganganagar, IND

**Keywords:** utm, cam, cad, gic, bopt

## Abstract

Background

Because all-ceramic crowns are more aesthetic and biocompatible than metal-ceramic crowns, they have grown in popularity among patients and dentists. Poor finish line layout can result in restoration margin fracturing, hence, finish line arrangement is critical to maintaining the restoration’s marginal integrity. The goal of this in-vitro study is to evaluate zirconia’s resistance to fracture (Cercon) ceramic restorations with three marginal designs (no finish line, heavy chamfer, and shoulder). This study is important in contributing to the ongoing debate about the optimal finish line design for zirconia restorations.

Methodology

Three different finish lines, namely, biologically oriented preparation technique (BOPT) with a marginal width of less than 0.3 mm, heavy chamfer with a marginal width of up to 0.3 mm, and shoulder with a marginal width greater than 0.3 mm, were made on 10 extracted maxillary first premolar tooth to make 30 epoxy resin dies on which zirconia (Cercon) coping was done using CAD/CAM technology, and marginal discrepancies were measured using a three-dimensional scanner. All the copings were affixed to their respective dies using GIC luting cement, and fracture resistance was measured using a digital universal testing machine.

Results

The Kruskal-Wallis test revealed that the mean fracture resistance was more in the heavy chamfer finish line, followed by the no finish line (BOPT) and the shoulder finish line. No statistically significant difference was seen between the no finish line and the heavy chamfer finish line. There was a significant difference between the heavy chamfer and shoulder finish lines (p = 0.004).

Conclusions

To increase the biomechanical performance of posterior single zirconia restorations, heavy chamfer margins are indicated.

## Introduction

Every tooth in the mouth is more precious than a diamond. Teeth that are severely fractured, have extensive fillings, are significantly discolored and do not react well to whitening techniques, and are aesthetically unappealing must be fixed with dental crowns. All-ceramic crowns are more aesthetic and biocompatible than metal-ceramic crowns, becoming progressively more popular among patients and dentists. They have a significant fracture rate, especially when utilized for posterior crowns. They are vulnerable to tensile loads and mechanical resistance due to exterior flaws and inner gaps. This is mostly due to the amount of biting stresses applied to posterior teeth, as well as the inherent brittleness of ceramics [1].

Zirconia has recently become the material of choice in modern restorative dentistry. It is the most fracture-resistant, all-ceramic material, and when used in CAD/CAM systems, it consistently enables the most beautiful, lifelike recreation of natural teeth [2]. It has received positive feedback from both dentists and patients. Poor finish line layout can result in restoration margin fracturing; hence, finish line arrangement is critical to maintaining the marginal integrity of the restoration. However, some zirconia crowns fracture mainly due to the magnitude of the biting forces applied on the premolar and molar teeth and due to the inherent brittleness of ceramics. The shoulder finish line which is used in all ceramics with 130 degrees has the advantage of beveling and having adequate preparation. Heavy chamfer finish line with more than 90 degrees cavo-surface line angle is used as it gives perfect marginal fit. In photoelastic experiments, low stress levels were observed in the chamfer finish line design, which could minimize stress-related cement failure [3]. The shoulder margin, on the other hand, may enhance the performance of single-crown alumina restorations biomechanically, according to a theory proposed by Di Lorio et al. [1]. The biologically oriented preparation method (BOPT) recently evolved from feature edge. When the clinical crown is employed, it is more conservative, and the anatomic crown does not match due to periodontal disease [4]. BOPT has clinical advantages such as the erasure of anatomical cementoenamel junction in unprepared teeth and deletion of the previously existing finish lines in prepared teeth.

The aim of this in vitro investigation is to assess the fracture resistance of zirconia (Cercon) ceramic restorations to three marginal designs (BOPT, heavy chamfer, and shoulder).

## Materials and methods

**Figure 1:**
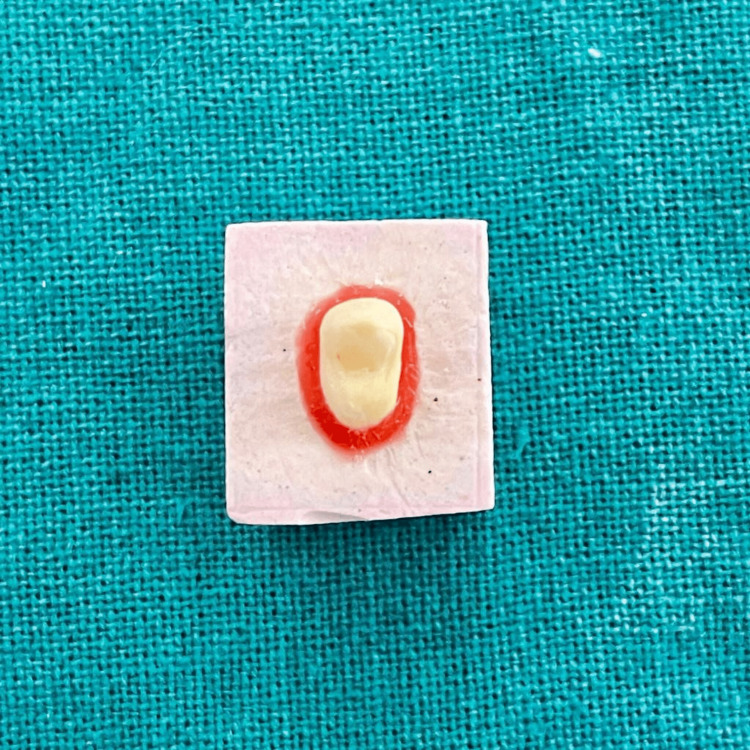
BOPT

For the current investigation, 10 undamaged, caries-free, first maxillary premolars removed for orthodontic purposes were preserved in a 10% formaldehyde solution (Vinayak Manutrade, India) for two weeks. All teeth were prepared in BOPT 1 mm below the cementoenamel Junction with no finish line using an 862F-012 - 120 microns grit flame-shaped bur (Coltene, Switzerland) with a width of less than 0.3 mm. The occlusal surface was prepared with a cusp form for increased strength resistance (Figure 1). Chemically activated polymethyl methacrylate (Pyrax, India) was used to make 10 special trays, and a polyvinylsiloxane impression material was used to make 10 imprints (Affinis Coltene, Switzerland).

**Figure 2:**
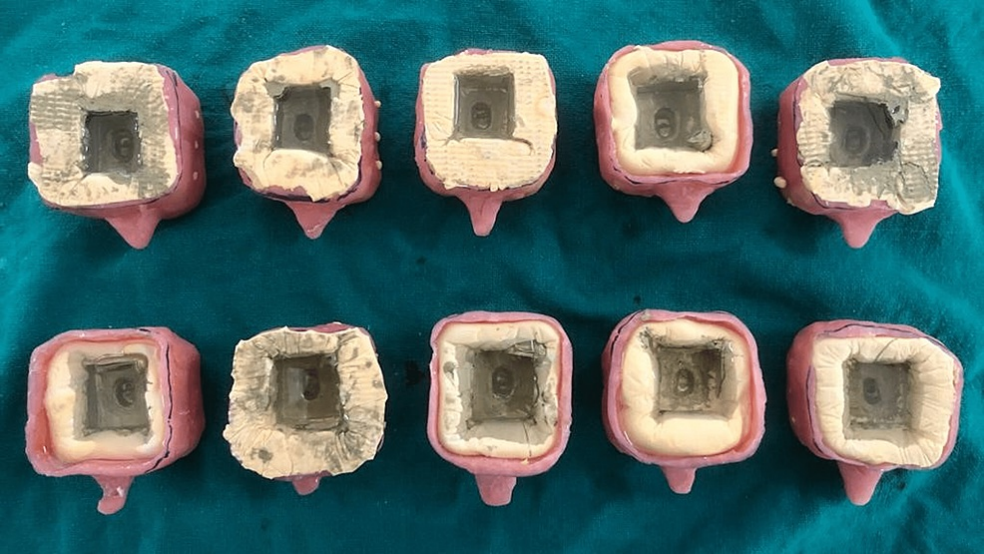
Ten Polyvinylsiloxane Impressions poured with Epoxy Resin and Hardener

**Figure 3:**
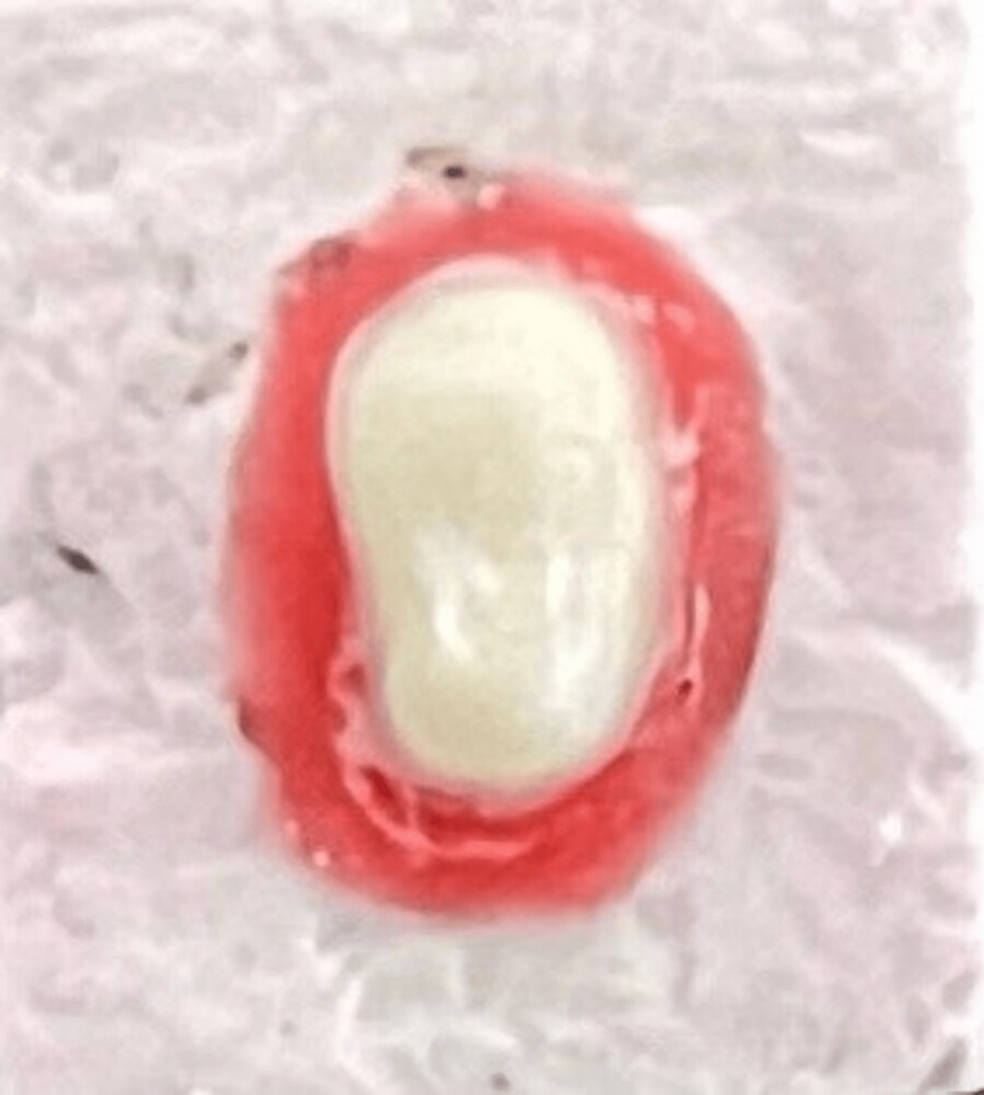
Heavy chamfer.

**Figure 4:**
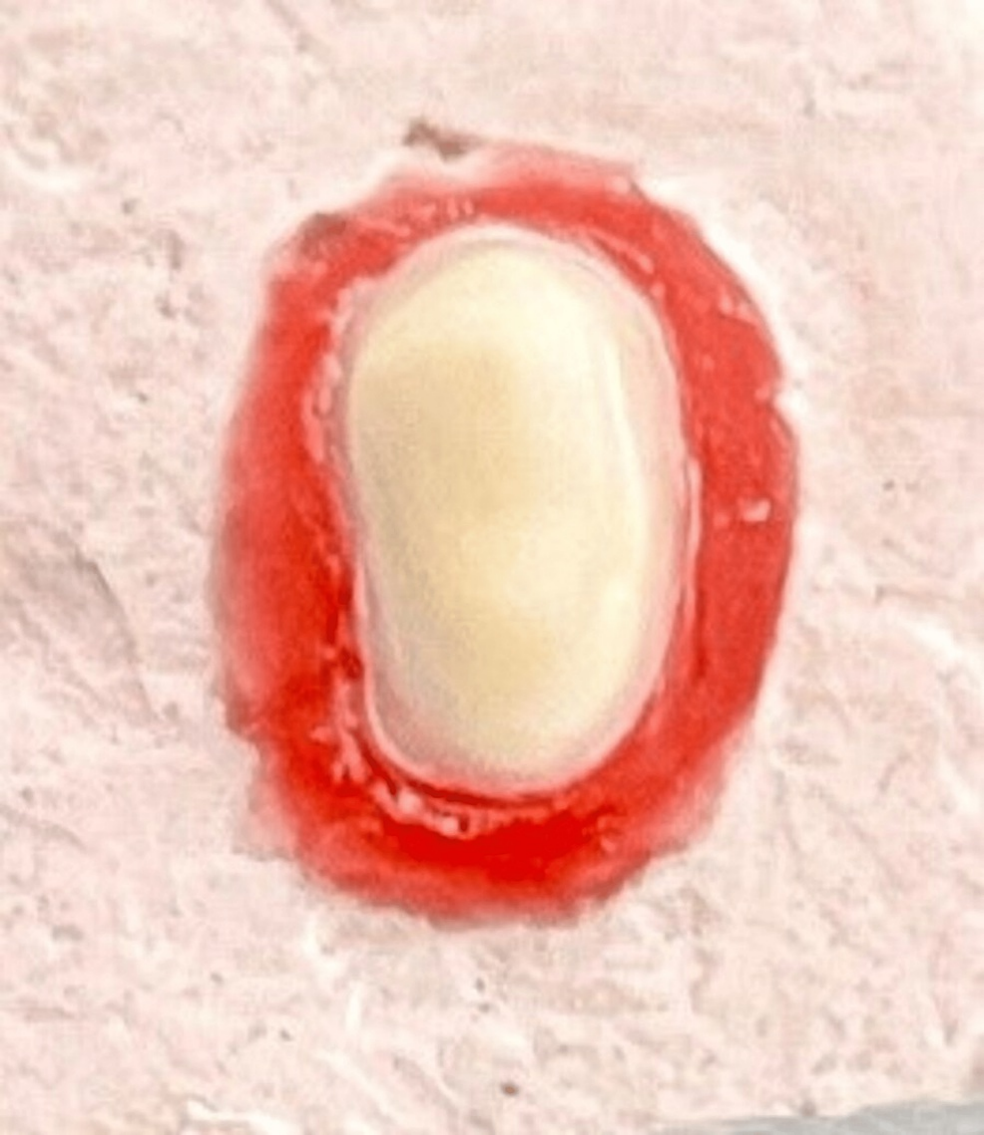
Shoulder.

To manufacture 10 identical resin dies with BOPT finish line margin, all 10 impressions (Figure 2) were poured using an ultra-clear epoxy resin and hardener (Haksons, India). The teeth were then extracted, and the BOPT margin was transformed into a heavy chamfer (Figure 3) with an 879/0126-Torpedo diamond bur (Coltene, Switzerland) with a width of up to 0.3 mm. Ten polyvinylsiloxane imprints were generated, and 10 epoxy resin dies with thick chamfer finish lines were manufactured from these impressions. With the 837-012 - cylindrical diamond bur, the thick chamfer finish line margin was turned into a shoulder (Figure 4) (Coltene, Switzerland) with a width greater than 0.3 mm. Ten polyvinylsiloxane impressions were obtained, which were then used to manufacture 10 epoxy resin dies.

**Figure 5:**
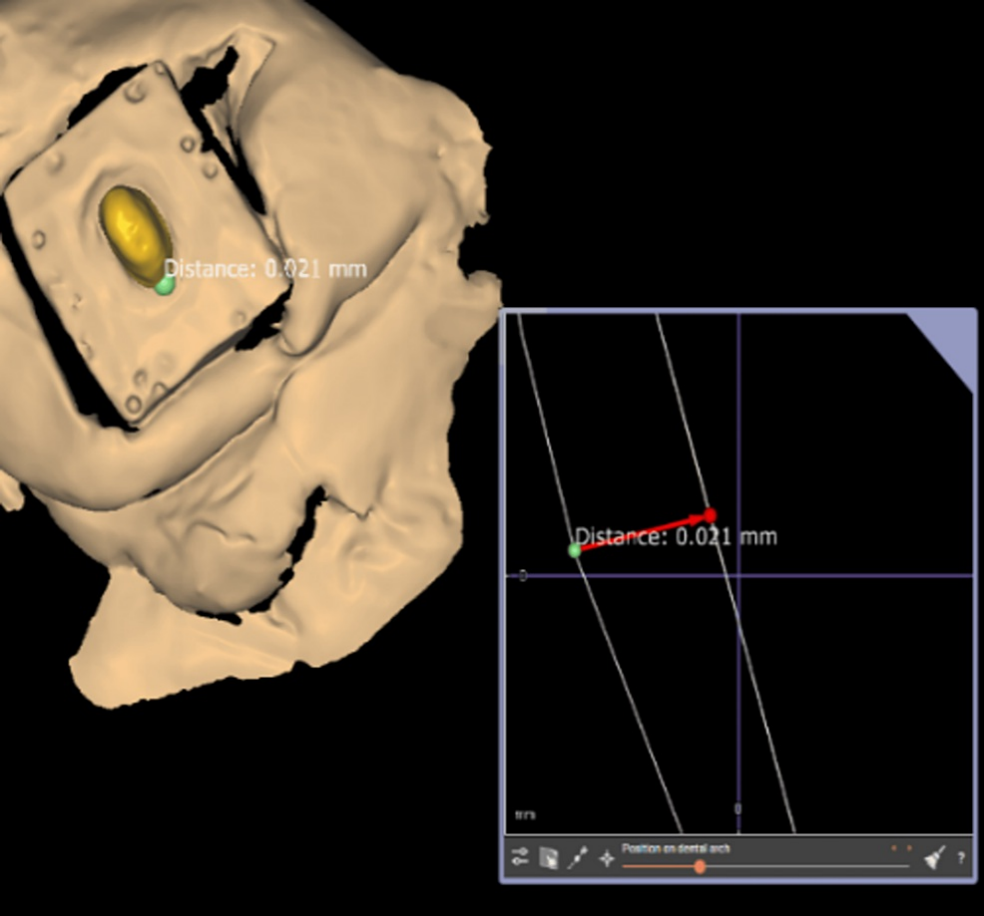
Marginal discrepancies evaluated using a three-dimensional scanner.

In total, 30 copings were manufactured in sintered ZrO_2_ ceramic material utilizing Cercon HT disc CAD/CAM (Scan 24 P53, China) technology (Dentsply, India) by marking the finish lines in exo-cad software. Copings with 0.5 mm thicknesses and 35 m of cement space were milled from pre-sintered ZrO_2_, and the Cercon copings were heat sintered for six hours at 1,530°C following the sintering schedule. The copings were tested for minor differences on their respective epoxy resin dies using a three-dimensional (3D) scanner (Shining 3D EX Pro, China) (Figure 5). GIC luting cement was then used to bond each coping to its final die (Shofu, Japan).

**Figure 6:**
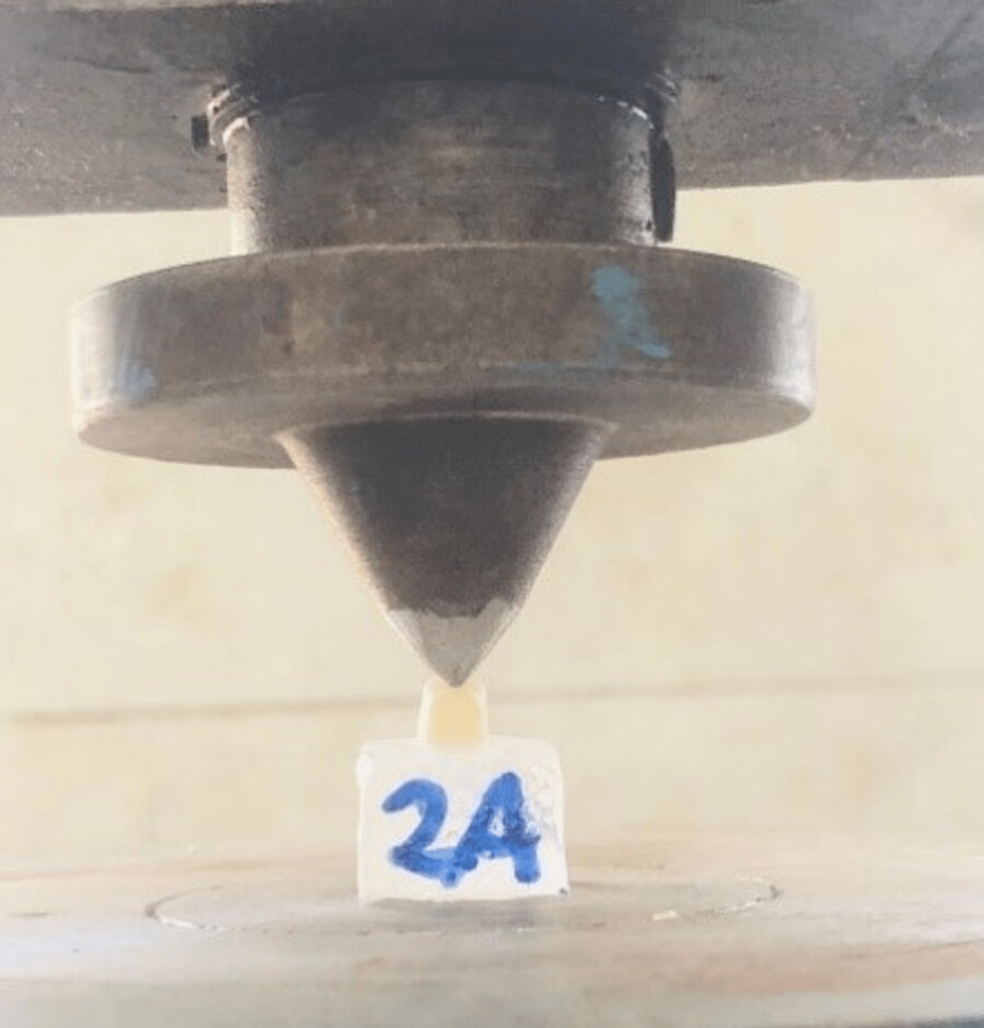
Fracture resistance performed using a digital universal testing machine.

During the setting time, finger pressure was applied. The extra luting cement was eliminated. After cementation, the samples were kept at room temperature in a saline solution (Denis, India). Fracture resistance was done by using digital universal testing equipment (ETTL Groups, India) (ETTL Groups, India). Along the long axis, the load was applied at the center of the occlusal surface with a 1 mm/minute crosshead speed, a 5 mm diameter stainless steel tip, and a 5 N minimum load until fracture occurred (Figure 6). Load controller software was used to automatically record the fracture load data. The samples were examined from the standpoint of the failure’s genesis. The data were examined using the Kruskal-Wallis test, and descriptive statistics of fracture resistance were calculated.

## Results

**Figure 7:**
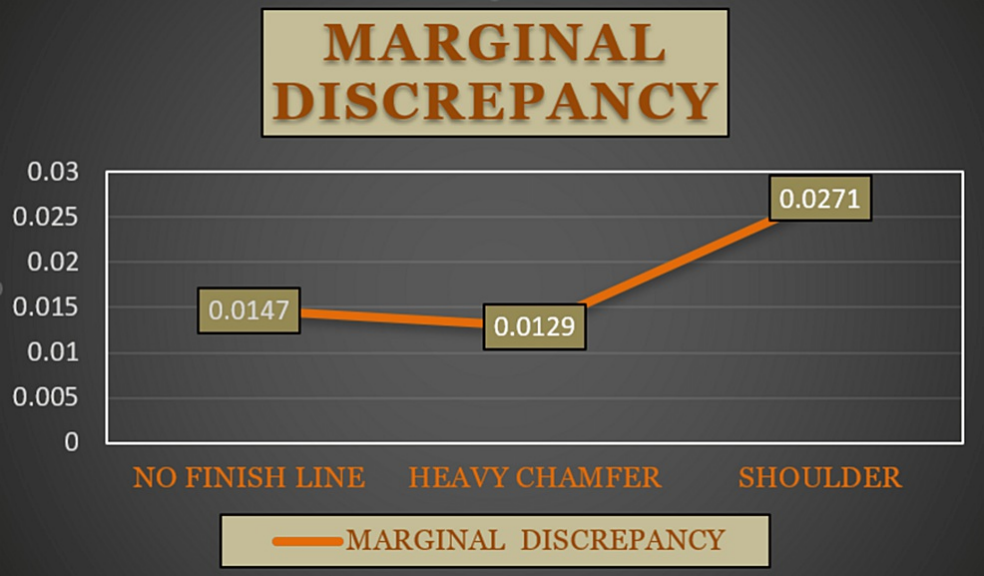
Line diagram showing the mean marginal discrepancies of the no finish line, heavy chamfer, and shoulder marginal designs.

**Figure 8:**
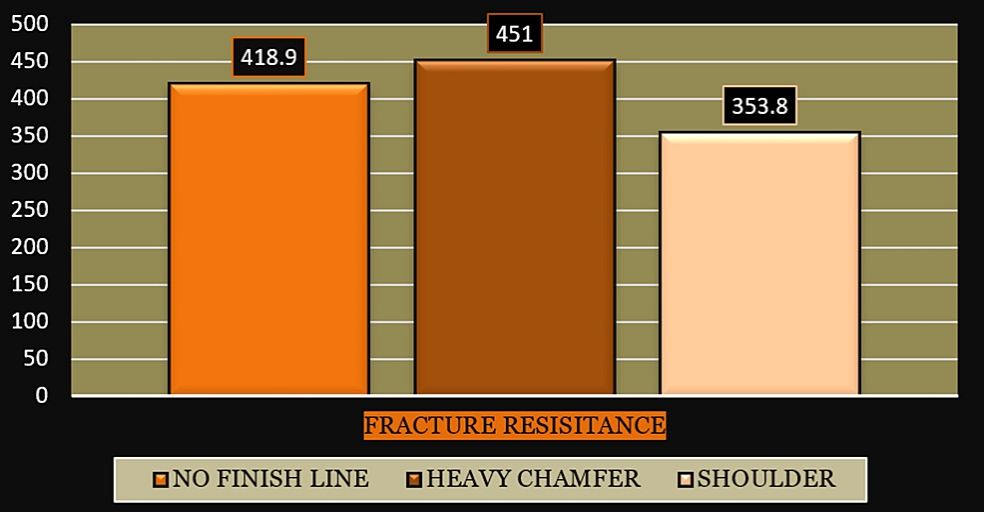
Bar diagram showing the mean fracture resistance of zirconia copings on the no finish line, heavy chamfer, and shoulder marginal designs.

In this study, the BOPT finish line had a mean marginal discrepancy of 14.7 µm, the heavy chamfer finish line had a mean marginal discrepancy of 12.9 µm, and the shoulder finish line had a mean marginal discrepancy of 27.1 µm (Figure 7). BOPT finish line had a mean fracture resistance of 418.90 N, the heavy chamfer finish line had a mean fracture resistance of 451.00 N, and the shoulder finish line had a mean fracture resistance of 353.80 N (Figure 8).

The mean and standard deviation of fracture resistance among no finish line, heavy chamfer, and shoulder designs was 418.90 ± 88.275, 451 ± 90.266, and 353.80 ± 64.327, respectively (Table 1). As the p-value was more than 0.001, there was no statistically significant difference between the heavy chamfer and no finish line designs. Given that the p-value was less than 0.001, there was a statistically significant difference between the heavy chamfer and shoulder finish line designs (Table 2).

**Table 1:** Descriptive statistics of fracture resistance

Study groups	Number	Minimum	Maximum	Mean	Standard deviation
No finish line	10	340	630	418.90	88.275
Heavy chamfer	10	341	650	451.00	90.266
Shoulder	10	302	490	353.80	64.327

**Table 2:** Pair-wise comparison of the means of fracture resistance between the three study groups (post-hoc Dunn’s test).

Study groups		Mean difference	P-value
No finish line	Heavy chamfer	-32.100	0.341
	Shoulder	65.100	0.052*
Heavy chamfer	No finish line	32.100	0.341
	Shoulder	97.200^*^	0.004*
Shoulder	No finish line	-65.100	0.052*
	Heavy chamfer	-97.200^*^	0.004*

## Discussion

Zirconia has recently become the material of choice in modern restorative dentistry. Cercon HT was utilized in this study. Kongkiatkamon et al. performed a comparative analysis of four popular translucent zirconia brands (AmannGirrbach, Cercon HT, Cercon XT, and Vita YZ XT) and concluded that Cercon HT had the best strength qualities and the largest fracture load [5]. The elastic modulus of the core’s supporting materials influenced its fracture resistance [6]. Epoxy resin dies have more elastic modulus than brass dies and are dimensionally more stable. Another distinction from clinical situations is the uncertain nature of the bonding between the luting agent and the die material. It is plausible to assume that the existence of a hybrid layer at the dentin-cement interface influences the biomechanical behavior of the core/supporting die system. However, because each of these factors had an equivalent impact on the samples in this investigation, a comparison between the three groups is conceivable.

The CAD/CAM technology with zirconia has the best fracture strength of any all-ceramic material and has consistently permitted the most attractive, lifelike recreation of natural dentition [2]. To reduce marginal differences and maximize fracture resistance, CAD/CAM technology was employed in this study. Moreover, due to higher stress concentrations at the margins and/or internal surfaces, there have been poor marginal and/or internal adaptations which might impact the fracture strength of restorations [7]. According to the American Dental Association, the film thickness for type I cement should not exceed 25 m, and 40 m for type II cement. Models with BOPT finish lines had a mean marginal difference of 14.7 m, models with heavy chamfer finish lines had a mean marginal discrepancy of 12.9 m, and models with shoulder finish lines had a mean marginal disagreement of 27.1 m in this study.

In this study, we compared three finish lines, with the shoulder finish line and heavy chamfer being the most commonly employed for zirconia restorations. Nankali [8] investigated chewing in a group of people. The maximal masticatory power in some people can reach 350 N. In this study, the load was put on the long axis at the center of the occlusal surface with a 1 mm/minute crosshead speed, a 5 N minimum load, and a stainless steel tip with a 5 mm diameter on all 30 cemented copings on respective dies until a fracture occurred, and the fracture load information was captured automatically using load controller software. Copings with BOPT finish lines had a mean fracture resistance of 418.90 N, copings with heavy chamfer finish lines had a mean fracture resistance of 451.00 N, and copings with shoulder finish lines had a mean fracture resistance of 353.80 N.

Kumar et al. [9] investigated the influence of the effect of the chamfer and shoulder on the resistance to fracture of all ceramic restorations in their study. These crowns demonstrated that the chamfer margin had higher mean fracture resistance than the shoulder margin. The Kruskal-Wallis test indicated that the mean fracture resistance among no finish line, heavy chamfer, and shoulder designs was higher in heavy chamfer (451.90.266), followed by no finish line (418.9088.275) and shoulder (353.8064.327). There was no discernible difference between no finish line and heavy chamfer. A substantial difference was noted between the heavy chamfer and shoulder finish lines, with a mean difference of about 97.200 and a p-value of 0.004.

Study limitations

The epoxy resin used for casting impressions had a long setting time of one to three days. Cementation with GIC was done by thumb-finger pressure. For fracture resistance, only compressive stresses were considered.

## Conclusions

Within the limitations of the present study and data obtained, it can be concluded that a heavy chamfer finish line is recommended to improve the biomechanical performance of posterior single zirconia restorations because of higher fracture resistance values.
